# A Colonial Congress

**Published:** 1889-03-09

**Authors:** 


### A Colonial Congress.

*(Concluded from p. 345.)*

After a week of pleasurable work and much excitement, the Medical Congress has drawn to an end, and all are returning to their homes with food for thought for the next three years, when they will meet again at Sydney, under the presidency of Dr. M'Laurin. Baron Von Mueller, who delivered an address on pharmacology at the last meeting, strongly recommended that the leading Universities of the world should be invited to send representatives to the next congress, which suggestion will probably be carried out.

A paper on the Treatment of Phthisis by Climate, by Dr. D. Turner, of Melbourne, was heard with much interest by all present. It was pointed out by Dr. Turner that while not a few still deny the curability of phthisis, the number of these sceptics is diminishing every year. At the present day the great bulk of the profession agree that consumptives are frequently restored to good sound health, and that the number of such cases would be much greater could patients be placed in the early stages of the disease under favourable climatic conditions. He thought it might fairly be doubted whether any drug, or combination of drugs, ever cured an ordinary case of lung phthisis when climatic conditions were not favourable. The high altitude treatment rested on a truly scientific basis. The following were the attributes of mountain air to which might be ascribed its curative properties in phthisis:—Purity, rarefaction and consequent low barometric pressure; coldness and absence of vegetation; dryness, intensity of light, stillness, absence of wind and much ozone. It was an undoubted fact that not only chest invalids, but even people in health who had lived in elevated localities for a few months gained considerable expansion of the lungs. Dr. Turner concluded by stating that he considers the ocean climate the best; after that, the high altitude climate at or above 3,000 feet; then the climate of the inland plains; and lastly, the climate of the marine resorts.

The question of typhoid which took up so much of the time of the Congress, can scarcely be so interesting to you in England as it is to us here. During last week the Melbourne Board of Health returns gave 69 new cases, and 23 deaths; so of course the subject is a burning one here. After much discussion it was agreed that typhoid was a preventible disease, and that our drainage is entirely to blame for its unpleasant frequency. Professor Allen was loudly applauded for stating with emphasis that in cases of typhoid if a good dose of calomel could be given during the first week, the process of continued poisoning might be prevented.

The hero of France, in the shape of M. Pasteur, received but scant courtesy from some of the members of the Congress. Dr. Wilkinson protested against carrying the inoculation theory so far as to introduce haphazard into the system poisons which might prove dangerous. Dr. Wigg read a paper on Pasteur's method of rabbit extermination, which he declared had turned out an absurd failure. He also affirmed, on the authority of Professor Peters, that the mortality in France from hydrophobia, which was formerly 31 per annum, had, since the invention and general use of his cure, been increased to over one hundred per annum. Whatever may be the value of these statements, it is apparent that inoculation as a mode of protection against various diseases cannot yet be regarded as the beneficent discovery which some practitioners were prepared to pronounce it. And in connexion with this subject the general public will be disposed to regret that no discussion took place at the Congress on the question of the legal enforcement of vaccination. The care and treatment of the insane was the subject of numerous papers, including one by Dr. Beattie Smith with special reference to the boarding-out system. Dr. Smith advocated the establishment of acute wards for the treatment of new cases, and chronic wards for general cases, and also suggested that criminal cases should only receive attention by the penal authorities, as the criminal patients were a disturbing element in asylums. As it was almost an indisputable fact that there was a large proportion of harmless insane, he considered that as far as possible they should be boarded out with suitable persons of the same religion and social class as the patient, and possessing the necessary qualifications to enable them to make periodical reports to the superintendent, who would every quarter examine the boarding out establishments and the patients. In Scotland this sys em had been found to be largely adopted with successful results, and the tendency generally was at present to avoid, if possible, the segregation of patients.

The following are the names of the English doctors who attended the Congress: Dr. Rankine Dawson, Dr. Hull, Dr. J. J. Powell, Dr. E. Thane, and Dr. R. Vassie.

